# Buccal Insulin Delivery Systems: Formulation Approaches, Stability Constraints, and Translational Prospects

**DOI:** 10.3390/pharmaceutics18091093

**Published:** 2026-08-30

**Authors:** Jahanvi Patel, Michael Stolinski, Anil Vangala

**Affiliations:** School of Life Sciences, Pharmacy and Chemistry, Faculty of Health, Science, Social Care and Education, Kingston University, Kingston Upon Thames, Surrey KT1 2EE, UK; k2247049@kingston.ac.uk (J.P.); m.stolinski@kingston.ac.uk (M.S.)

**Keywords:** insulin delivery, buccal administration, mucoadhesive systems, nanoparticles, deformable vesicles, permeation enhancement, protein therapeutics

## Abstract

Insulin delivery via the buccal route has shown long-standing promise for diabetes therapy, but clinical translation remains limited. Most systems address only part of the delivery problem, whereas a viable product must simultaneously provide adequate insulin residence time, effective mucus and epithelial permeation, stability, dose consistency, safety, and manufacturability. This review examines the biological and formulation barriers limiting buccal insulin delivery and compares major platform types, including mucoadhesive films and patches, nanocarrier-based systems, deformable vesicles, chemistry-led permeation approaches, and device-enabled strategies. Stability, alongside limited permeability, is considered a key barrier, while aggregation and excipient-related instability remain insufficiently addressed. This review also discusses why promising preclinical findings often fail to translate, exhibiting low bioavailability, variability, safety concerns, and scale-up challenges recurring across platforms. Overall, further progress in buccal insulin delivery will depend on formulation strategies that better integrate permeation enhancement, stability preservation, and translational feasibility.

## 1. Introduction

Diabetes mellitus remains one of the most prevalent chronic metabolic disorders worldwide, and insulin therapy is indispensable for all patients with type 1 diabetes and for many with progressive or advanced type 2 diabetes [1,2]. Although subcutaneous administration remains the clinical standard, repeated injections still impose pain, needle aversion, treatment burden, and injection-related complications, sustaining interest in noninvasive insulin delivery strategies [2,3,4,5]. Alternative routes, including oral, buccal, nasal, and transdermal delivery, have therefore been explored to improve adherence and reduce dependence on injections [4,5]. Among these, buccal delivery has drawn longstanding interest because it provides direct access to a vascularized mucosa while avoiding gastrointestinal degradation and hepatic first-pass metabolism [6,7,8].

**Figure 1:**
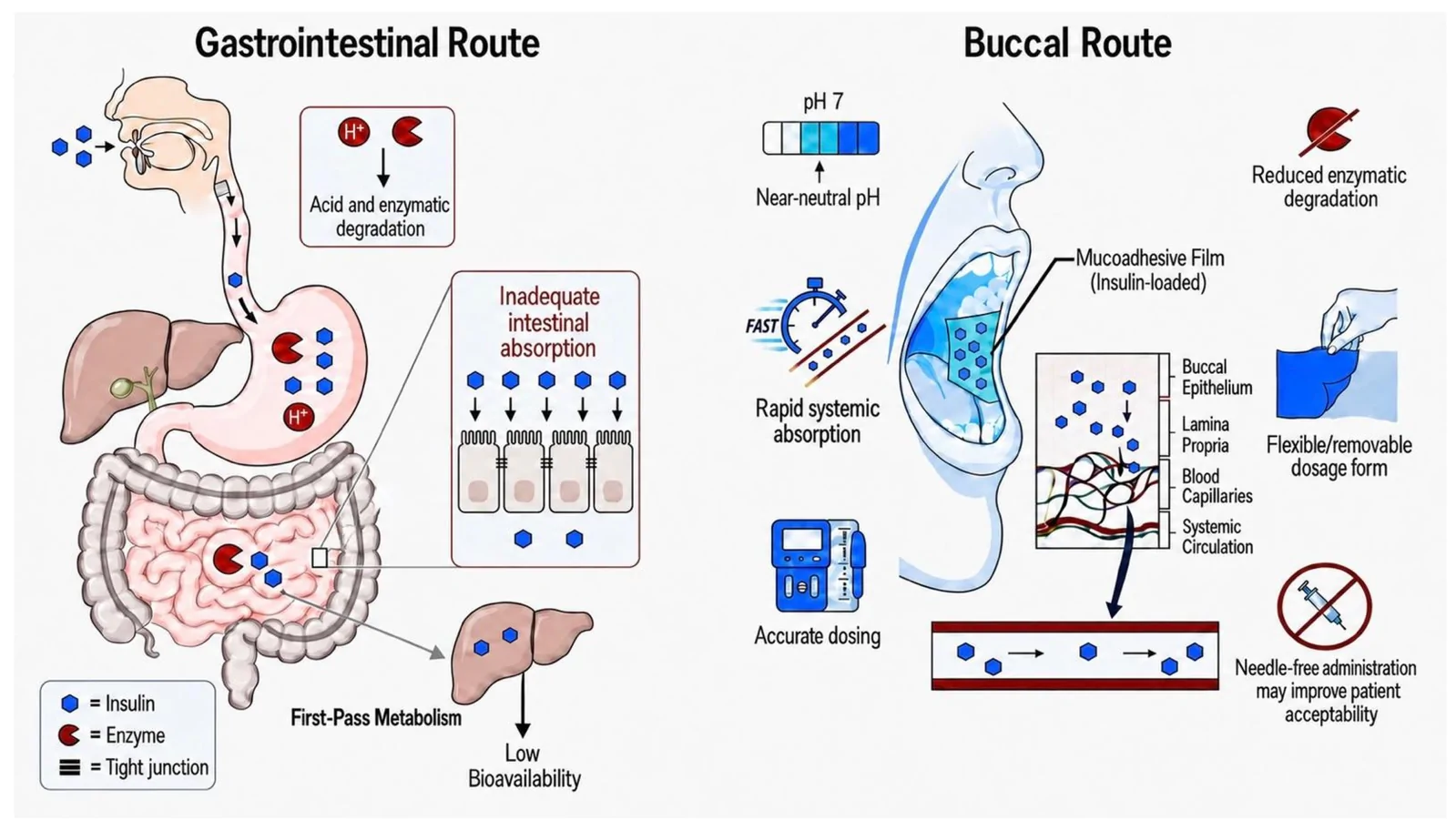
Comparison of gastrointestinal and buccal routes for insulin delivery. Source: Figure created using Figure Labs, AI Scientific Figure Illustrator (https://www.figurelabs.ai/, accessed 30 July 2026).

Buccal delivery is also pharmaceutically attractive because the oral cavity offers a near-neutral environment with lower enzymatic activity than the gastrointestinal tract, making it more favourable for structurally fragile peptides such as insulin [2,6]. Buccal films and patches can be designed as flexible, patient-friendly dosage forms with accurate unit dosing and the practical advantage that administration can be stopped by removing the system if needed. From a patient perspective, a buccal film or patch needs to remain attached to the inner cheek for the required dosing period. Saliva, swallowing, speaking, and chewing may shorten contact time or dislodge the formulation. Depending on the design, patients may therefore need to avoid eating or drinking while it is in place. Acceptability may also depend on taste, mouthfeel, stickiness, thickness, and any local irritation [9,10]. Although thin, flexible films are intended to minimise discomfort, these sensory and practical factors should be considered during formulation development [9,11]. A comparison of buccal and gastrointestinal routes of insulin delivery is illustrated in Figure 1. Oral transmucosal films are already established in clinical practice. Marketed products such as Belbuca^®^, which delivers buprenorphine for chronic pain, and Libervant^TM^ are labelled as buccal films that deliver diazepam for the acute treatment of seizure clusters, supporting the translational feasibility of the dosage form itself, although the approved products are mainly intended for potent small molecules rather than large hydrophilic peptides such as insulin [9,12]. Early human studies with buccal and oral insulin spray suggest that the route may support relatively rapid systemic action, which is especially relevant for prandial insulin delivery [13,14,15]. Even so, buccal delivery remains challenging because insulin crosses the buccal mucosa very poorly. Systemic delivery is limited by the nonkeratinized stratified epithelium, intercellular lipids, mucus, local enzymatic activity, limited absorptive area, and continuous salivary clearance [6,9,16]. These barriers are especially important for insulin, a large hydrophilic peptide whose passive transport across intact buccal mucosa is intrinsically inefficient [16].

Despite these constraints, clinical proof of concept has been achieved. The Oral insulin spray, delivered using the Rapid Mist device, showed that insulin delivered to the oral mucosa could produce measurable pharmacokinetic and pharmacodynamic effects, including faster onset and shorter duration than subcutaneous insulin [13,14]. However, later analyses also showed why this early success did not translate into durable clinical use, with low relative biopotency, high dose burden, and marked variability emerging as key limitations [7,17]. These findings shifted the field toward broader formulation innovation, including mucoadhesive films and patches for residence control [9,18,19], unidirectional buccal tablet systems for directional release [20], nanoparticle-based and nanoparticle-in-film systems for insulin protection and mucosal interaction [10,21,22], deformable vesicles and related lipid carriers for enhanced transport [23,24,25,26], and chemical enhancement strategies including hydrophobic ion pairing, cell-penetrating peptides, and ionic liquids for improved permeation [27,28,29]. In recent years, device-based buccal delivery approaches have emerged as an additional strategy to reduce reliance on passive permeability by actively overcoming the epithelial barrier, including microneedle-mediated penetration that deposits insulin directly into the buccal mucosa and suction-assisted systems that stretch and thin the epithelium, increasing transport of macromolecules and, in some designs, being combined with permeation enhancers to further improve delivery [30,31,32].

Another challenge, often treated as secondary despite its translational importance, is insulin stability. Insulin is structurally sensitive to unfavourable pH, thermal stress, agitation, contact with container surfaces, and hydrated formulation environments, all of which can promote conformational change, aggregation, and loss of biological activity [33,34,35]. In buccal delivery, this issue extends beyond storage because insulin may also be exposed to destabilising processing conditions, salivary dilution, and excipient environments before absorption occurs [19,26,36]. Buccal insulin delivery should therefore be viewed as a combined formulation, permeability, stability, and translational problem rather than a simple permeability challenge. This review examines the field from that integrated perspective of buccal insulin delivery, covering mucoadhesive films and patches, nanocarrier and vesicular systems, permeation-enhancing formulations, and device-based approaches. It focuses on how formulation design, insulin stability, and practical translational challenges must be addressed together to develop clinically useful systems.

## 2. Barriers to Buccal Insulin Delivery

**Figure 2:**
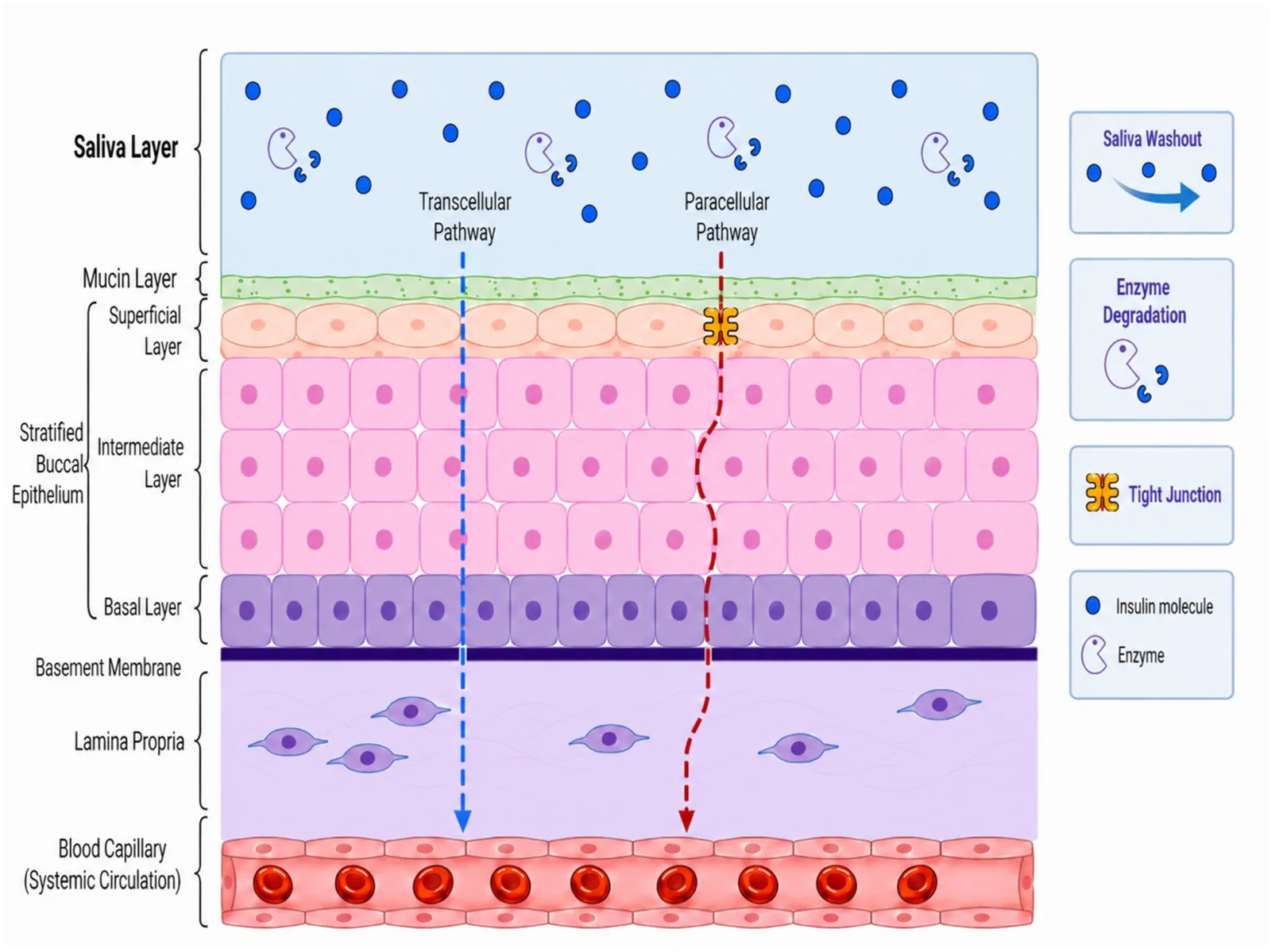
Major physiological barriers to buccal insulin delivery. Source: Figure created using Figure Labs, AI Scientific Figure Illustrator (https://www.figurelabs.ai/, accessed 30 July 2026).

For insulin, the main attraction of buccal delivery is that it avoids the harsh gastrointestinal environment and first-pass metabolism in the liver, both of which make conventional oral delivery difficult [2,6,37]. This is especially important for protein and peptides that are easily degraded in the gastrointestinal tract and therefore usually need to be administered by injection. This gives the route a clear pharmacokinetic advantage, but it does not mean that buccal absorption is easy. As summarised in Table 1, buccal insulin delivery is limited by interactions with various insulin-related physicochemical, physical, physiological, and enzymatic factors. Figure 2 shows the key epithelial barriers that limit buccal insulin delivery.

Insulin self-association is an important factor when considering barriers to buccal insulin delivery. Insulin is a self-associating protein, and its apparent hydrodynamic size depends on formulation and dilution conditions. Insulin transport across the buccal epithelium is influenced by its association state. Gast et al. [41] reported a Stokes radius of approximately 1.33–1.45 nm for largely monomeric insulin after dilution, whereas insulin in the initial pharmaceutical formulation exhibited larger radii of approximately 2.5–2.7 nm, consistent with compact hexameric assemblies. Additionally, considering the 1.5–3 nm pore radius range summarised in Table 1, insulin transport may vary with association state, and larger oligomeric species are likely to experience greater steric hindrance than monomeric insulin. Thus, understanding and controlling insulin self-association is essential for developing effective buccal insulin delivery systems.

## 3. Formulation Strategies for Buccal Insulin Delivery and Their Limitations

**Figure 3:**
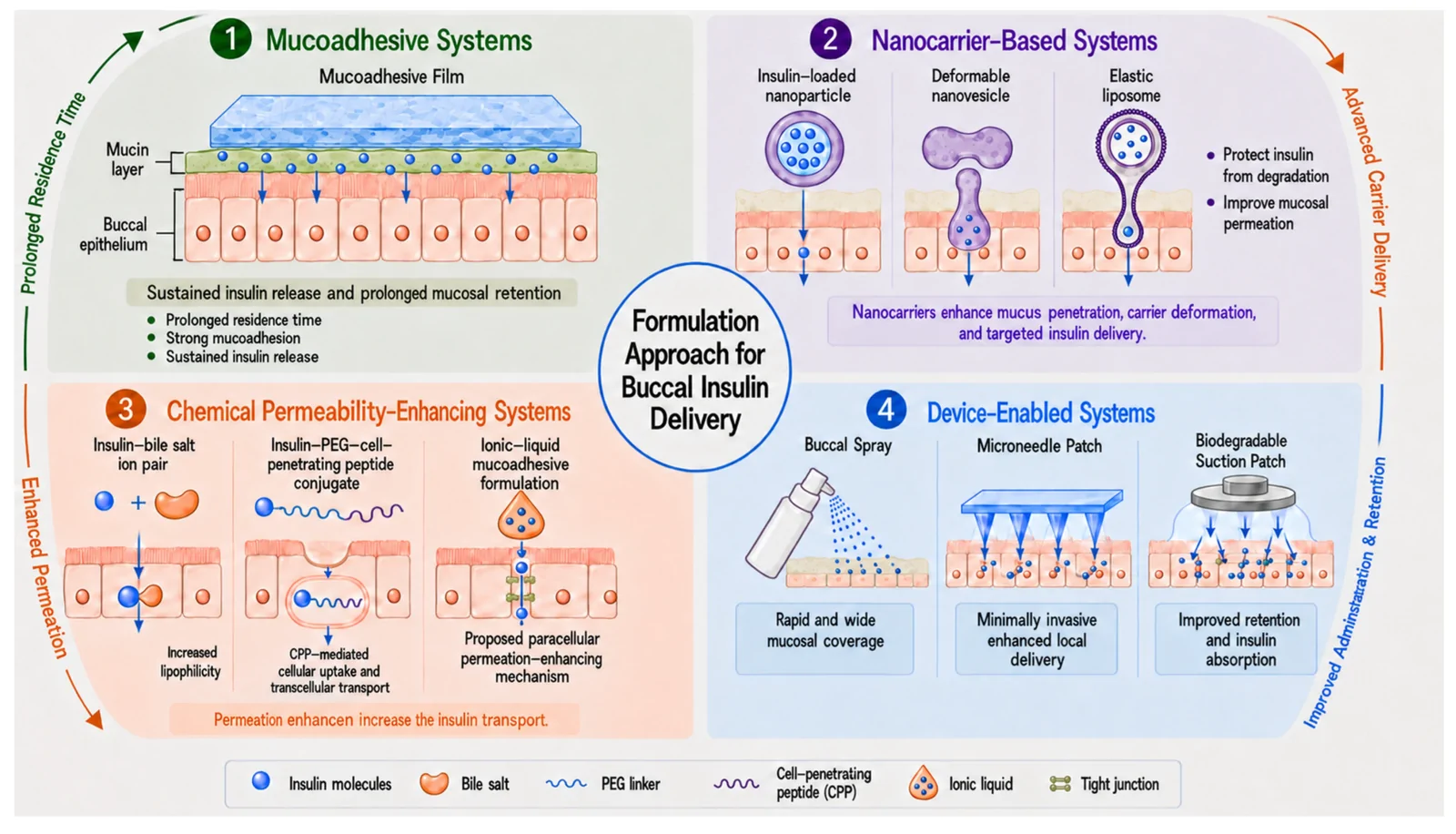
Major formulation strategies for buccal insulin delivery. Source: Figure created using Figure Labs, AI Scientific Figure Illustrator (https://www.figurelabs.ai/, accessed 30 July 2026).

To improve buccal insulin delivery, researchers have developed several formulation strategies to overcome poor mucosal permeability, short residence time, and low dose efficiency [2,6,39]. These approaches vary in design, but most aim to either keep insulin at the mucosal surface, enhance its transport across the epithelium, or combine both functions within a single platform [7,10,22]. As shown in Figure 3, different formulation- and device-based strategies have been explored to improve buccal insulin delivery. Although they differ in approach, all designs aim to retain insulin at the mucosal surface, promote passage across the epithelium, and enhance systemic uptake despite normal buccal mucosal barriers.

### 3.1. Mucoadhesive Films and Patches

Mucoadhesive film systems are useful because they can combine several functions in a single platform, including prolonged mucosal residence, reduced salivary washout, closer contact with the absorption site, and, in some designs, unidirectional release toward the buccal tissue [9,18]. Their main advantage is that they reduce salivary washout and extend contact with the absorptive mucosa, creating better conditions for insulin absorption. As shown in Table 2, the different mucoadhesive film and patch approaches studied for buccal insulin delivery are summarised by design, preparation method, evaluation approach, key findings, and main limitations.

In one study, Chen et al. [18] reported an optimised bilayer film composed of a backing layer and a mucoadhesive insulin-loaded layer. This system improved adhesion-supported release toward the absorption surface and showed prolonged adhesion time, thereby promoting insulin absorption. Figure 3 illustrates this concept. Additionally, Table 2 summarises these findings. This approach increased insulin exposure compared with free insulin and maintained glucose control for about 8 h in diabetic rats [18]. These findings suggest that effective film performance depends on more than mucoadhesion alone and may improve when prolonged residence, controlled release, and permeation support are combined within the same system. However, this study did not measure the insulin association state, so the increased transport cannot be attributed to insulin dissociation and may instead result from prolonged adhesion, controlled release, or protamine-mediated permeation. Also, the evidence remains limited to preclinical studies.

In another study, Diab et al. [19] investigated a chitosan-based mucoadhesive buccal film incorporating insulin, glycerin, and L-arginine. As mentioned in Table 2, the formulation was characterised in terms of film appearance and flexibility, insulin content using ELISA, spectroscopic properties, and in vivo glucose-lowering efficacy. The optimised film retained at least 80% of the theoretical insulin content and produced an approximately 40% reduction in blood glucose levels in rats, corresponding to about 57% of the pharmacological effect achieved with subcutaneous insulin [19]. These results demonstrate measurable buccal insulin activity, although the overall efficacy remained lower than that of the injectable route. In this respective study, SRCD and FTIR-MS analysis findings suggested that L-arginine helped preserve insulin secondary structure, promoted greater preservation of insulin α-helical structure, and reduced these non-native spectral signals. However, this study did not report on insulin’s oligomeric state nor examine its transport across buccal tissue using an ex vivo permeation assay. As such, the glucose-lowering effect appears to be due to insulin stabilisation, longer film contact with the mucosa, and L-arginine’s permeation-enhancing effect. Further studies are needed to clarify the relationship between insulin oligomerisation and buccal permeation and to improve dose efficiency and overall therapeutic performance.

A different study proposed a unidirectional buccal tablet concept based on a triple-layer mucoadhesive system, in which nanoparticles loaded with insulin were added as dry powder to a multilayer mucoadhesive system [20]. As mentioned in Table 2, this unidirectional approach is proposed to minimise saliva dilution and drug loss, direct insulin release towards the buccal absorption site, and improve the stability of the dosage form. While this work did not progress beyond the concept stage, no experimental characterisation was performed. This study focused on conceptual design, multilayer configuration, and polymer selection. It demonstrates the importance of combining mucoadhesion, directional release, nanoparticle delivery, and enhanced stability in a buccal insulin delivery system.

Overall, studies show that mucoadhesive films, patches, and multilayer buccal systems can enhance mucosal residence, reduce salivary washout, and promote insulin release towards the absorption surface. Chitosan-based films demonstrated measurable in vivo glucose-lowering activity, while bilayer films increased insulin adhesion and exposure. Multilayer tablet concepts can also minimise drug loss through saliva and promote unidirectional release of the drug. Taken together, these findings may lay the foundation for integrated buccal insulin delivery platforms with mucoadhesion, controlled release, permeation support, and dosage-form stability.

### 3.2. Nanocarrier-Based Systems

Nanocarrier-based systems have emerged as a key strategy for improving buccal insulin delivery. Their main purpose is to protect insulin within the formulation, improve its interaction with the buccal barrier, and increase transport through carrier flexibility or an altered drug state [7,10]. Figure 3 illustrates this concept. Table 3 summarises different nanocarrier-based systems investigated for buccal insulin delivery, including nanoparticle-loaded films, deformable vesicles and elastic lipid-based carriers. It also highlights their compositions, preparation methods, and key optimised parameters, including particle size, encapsulation efficiency, release profile, permeability, relative bioavailability, and storage stability.

#### 3.2.1. Nanoparticle-Loaded Buccal Films

Nanoparticle-loaded buccal films were explored as hybrid systems that combine the retention advantage of mucoadhesive films with the protective and transport-supporting functions of nanoparticles. As outlined in Table 3, early work showed that insulin-loaded PEG-b-PLA nanoparticles could be incorporated into chitosan films with acceptable formulation properties, including a particle size of around 215 nm, an encapsulation efficiency of about 71%, and improved film flexibility, supporting the feasibility of this design [21]. This study did not directly assess the insulin association state after nanoparticle encapsulation and incorporation into the film. However, the physicochemical properties of the nanoparticles were influenced by the initial insulin loading. The formulation containing 2% *w*/*w* insulin loading showed the highest encapsulation efficiency. Increasing the insulin concentration led to lower encapsulation efficiency and the formation of large aggregates at 10% *w*/*w* loading. These aggregates resulted from emulsion destabilisation. They suggested that the PEG blocks in PEG-b-PLA may provide a steric barrier, reducing interactions between insulin and the PLA matrix during nanoparticle formation. Even so, they did not confirm this mechanism by directly assessing insulin’s association state or molecular conformation.

Morales et al. [22] prepared insulin-coated nanoparticles within polymeric films as a possible system for buccal delivery. The insulin-coated nanoparticles were dispersed in Eudragit RLPO, both alone and in combination with hydroxypropyl methylcellulose. They showed measurable transport across a human buccal model. All formulations were evaluated for nanoparticle characteristics, dry-film morphology, mucoadhesive properties, insulin release, storage stability, and permeation across a three-dimensional human buccal tissue model. The ERL film performed much better than the ERL-HPMC film, reaching about 52% cumulative insulin permeation over 4 h compared with about 10% for the blended film, with corresponding flux values of 0.34 ± 0.05 and 0.07 ± 0.02 μg/h/cm^2^, respectively. Taken together, these findings suggest that ICNP-loaded ERL films could provide a promising approach for buccal insulin delivery by combining particle stability, favourable film properties, and enhanced mucosal permeation. Nevertheless, further ex vivo and in vivo studies are needed to determine whether these encouraging in vitro results can translate into safe and effective insulin delivery.

#### 3.2.2. Deformable Vesicle Systems

Deformable vesicle systems were developed to improve buccal insulin transport by employing flexible lipid carriers that interact more effectively with the mucosal barrier than conventional vesicles. This is illustrated in Figure 3. As highlighted in Table 3, this approach evolved across nanocarrier studies, with differences in vesicle composition and preparation method affecting both delivery performance and formulation-specific behaviour. Yang et al. [23] compared deformable vesicles containing SPC, cholesterol, and sodium deoxycholate with conventional vesicles lacking sodium deoxycholate. The deformable vesicles produced greater pharmacological and pharmacokinetic bioavailability and higher residual insulin deposition in rabbit buccal tissue. These effects occurred despite slightly lower insulin entrapment efficiency than in conventional vesicles, suggesting that improved delivery related to vesicle properties rather than simply the amount of insulin entrapped. The authors proposed that vesicle deformability, hydration-driven penetration, and fusion with the buccal membrane contributed to the enhanced delivery. However, they did not evaluate whether vesicle formation or sodium deoxycholate altered insulin’s monomer–dimer–hexamer distribution, aggregation, or molecular conformation. Therefore, this study supports a formulation-dependent improvement in buccal insulin delivery but does not establish a direct relationship between insulin association state and transmucosal permeation rate as mentioned in Table 3. These findings provide direct in vivo evidence of buccal insulin delivery in rabbits. However, the proposed mechanisms involving vesicle deformability, hydration-driven penetration, membrane perturbation, and vesicle fusion were not directly measured.

In another study, insulin–phospholipid complex deformable nanovesicles were developed using lecithin, Tween 20, and sodium deoxycholate, showing that this design could support both insulin encapsulation and delivery performance [24]. In a reported study, ex vivo permeation across excised porcine buccal mucosa showed that IPC-DNVs produced the highest apparent permeability coefficient after 4 h. The 15.53% relative pharmacological bioavailability was obtained separately from the in vivo rabbit study. additional researcher further examined how insulin–phospholipid complexation and nanovesicle deformability influenced insulin structure and buccal transport. Circular dichroism analysis showed changes in the apparent α-helical content of encapsulated insulin, while insulin released from the nanovesicles retained a secondary-structure profile generally similar to free insulin. The authors suggested that the higher α-helical content observed in some formulations might be related to the formation of micelle-like structures or insulin aggregates. However, this possibility was not confirmed using direct analyses of insulin oligomerisation or aggregation. Therefore, the study supports a relationship between phospholipid complexation, nanovesicle deformability, and buccal insulin transport, but it does not prove that a specific insulin association state directly controls the transmucosal permeation rate.

In a subsequent work, Guo et al. [26] incorporated a comparable deformable nanovesicle system into a gelatine-based matrix. This formulation was more stable during refrigerated storage at 4 °C for up to 3 months and still maintained good delivery performance after storage, with no major changes in particle size, pH, or drug content and with preserved hypoglycaemic activity. The relative pharmacological bioavailability of 14.90 ± 3.12% was measured separately in the in vivo rabbit study. However, the study did not directly assess insulin association, aggregation, or conformational changes. Although the authors suggested that the gelatine network may help prevent vesicle fusion and preserve insulin structure, the findings do not demonstrate a direct link between insulin’s molecular state and its transmucosal permeation. Instead, the results indicate that the formulation itself influences insulin stability and transport. Taken together, this finding suggests that deformable vesicle systems are among the more promising passive nanocarrier approaches because they combine transport enhancement with formulation support and storage stability, although stronger clinical evidence is still needed.

#### 3.2.3. Elastic Liposomes

Elastic liposomes represent a related vesicular strategy in which lipid bilayers are modified with edge activators to increase flexibility, protection, and support buccal transport. Figure 3 illustrates this concept. As mentioned in Table 3, this approach moved from cell-based testing to ex vivo tissue evaluation. In TR146 cells, cholic acid derivative-modified elastic biosomes prepared by thin-film hydration showed particle sizes of about 140–150 nm. In the in vitro TR146 human buccal epithelial cell model, SDGC-modified elastic bilosomes produced the greatest insulin transport. SDGC-lipo showed a 5.24-fold enhancement in the permeability coefficient compared with insulin solution, while SC-lipo and SDTC-lipo showed 3.20- and 3.10-fold enhancements, respectively. Flow cytometry and confocal microscopy also showed the highest cellular uptake for SDGC-lipo. The TR146 cell layer integrity was maintained, with more than 96% TEER recovery after the experiment. No in vivo or ex vivo tissue study was performed [42]. This finding was strengthened further in ex vivo porcine buccal tissue, where sodium glycodeoxycholate-containing elastic liposomes showed an increase in permeability coefficient relative to insulin solution and performed better than sodium cholate liposomes [25]. The researcher investigated how the bile salt edge activator, sodium cholate or sodium glycodeoxycholate, influenced elastic liposome properties and insulin transport across porcine buccal mucosa. The SGDC formulation produced a 4.33-fold increase in the permeability coefficient compared with the insulin solution, while confocal microscopy showed stronger and deeper insulin distribution in the tissue than the other formulations. These findings suggest that edge activator chemistry and vesicle deformability contributed to enhanced buccal transport. However, the study did not assess insulin association state, aggregation, or molecular conformation [25]. Therefore, it supports a formulation-based explanation for insulin permeation but does not establish that changes in insulin association state caused the observed transport difference. Together, these results suggest that bilayer flexibility and bile salt modification can improve buccal insulin transport.

Overall, the nanocarrier-based approaches indicate that these systems contribute more than simply prolonging residence time. They also protect insulin from degradation and enhance carrier interaction with the buccal barrier, thereby improving delivery performance. Nanoparticle film systems primarily support formulation feasibility, whereas deformable vesicles and elastic liposomes exhibit greater transport-enhancing potential due to carrier flexibility and bilayer adaptability [21,22,23,24,25,26,42]. Even so, these advances show that nanocarrier design is moving in a useful direction, particularly in improving insulin protection and carrier interaction with the buccal barrier. Their future value will depend on how successfully these formulation strengths translate into buccal insulin platforms that are stable, reproducible, practical, and scalable.

### 3.3. Chemistry-Led Permeation Strategies

Chemistry-led permeation strategies were developed to improve buccal insulin delivery by changing the physicochemical behaviour of insulin or the microenvironment at the mucosal surface. Unlike films or vesicles, which primarily enhance residence time or carrier-mediated transport, these approaches act more directly on epithelial passage by increasing insulin lipophilicity, modulating barrier interactions, or promoting epithelial uptake [27,28,29]. Figure 3 provides a visual representation of this concept. Earlier sublingual work also showed that enhancers such as chitosan, hydroxypropyl-*β*-cyclodextrin, egg lecithin, oleic acid, and polyoxyethylene lauryl ether could alter mucosal barrier properties and insulin association state, helping explain the continued interest in chemistry-led strategies for buccal peptide delivery [43]. Table 4 compares the main chemistry-led strategies explored for buccal insulin delivery, focusing on how they improve mucosal transport, in vitro, ex vivo, or in vivo evaluation and the specific experimental model, the primary permeability or bioavailability outcomes reported, and the key limitations that continue to limit their translation into clinical practice.

#### 3.3.1. Hydrophobic Ion Pairing

Hydrophobic ion pairing was used to improve buccal insulin transport by forming complexes between insulin and bile salt derivatives, thereby increasing the molecule’s lipophilicity. Figure 3 presents this concept. One study investigated TR146 cell layers to assess epithelial transport and ex vivo porcine buccal tissue to examine permeation in a more physiologically relevant mucosal model, as shown in Table 4, with improved permeability in both systems relative to insulin solution [27]. The authors complexed insulin with sodium glycodeoxycholate to make it lipophilic. They found that transport of this complex was higher in TR146 buccal cells and ex vivo porcine buccal tissue than the insulin solution. What makes this approach especially interesting is that the improved transport was not accompanied by obvious acute damage in the TR146 model, since cell viability remained essentially unchanged and TEER (transepithelial electrical resistance) recovery stayed above 96% after exposure. One important limitation is that the HIP complex was formed and tested under different pH conditions. It was prepared below the insulin isoelectric point, where positively charged insulin can interact with negatively charged SGDC. However, the permeation studies were performed at pH 7.4, where both components are expected to carry negative charges. Although the complex showed higher lipophilicity and a near-neutral zeta potential, the study did not investigate whether it remained intact or dissociated at pH 7.4. Its stability during buccal permeation remains uncertain, highlighting an important gap in the physicochemical and translational understanding of HIP-based peptide delivery. These findings support the value of changing insulin’s physicochemical state to improve buccal transport. However, the study did not assess whether HIP complexation altered insulin’s association state. Even so, the evidence remained limited to in vitro and ex vivo evaluation and was not extended to an in vivo buccal dosage form [27].

#### 3.3.2. Ionic Liquid Systems

Ionic liquid systems were explored using choline geranate (CAGE) to improve buccal insulin transport, support the local formulation environment and promote possible paracellular transport mechanisms [29]. In this study, the researchers investigated the physicochemical properties of a CAGE-based buccal patch, including gel rheology, insulin loading, release, tissue transport, and in vivo activity. The patch preserved a high amount of active insulin and provided sustained release and buccal transport. The authors suggested that CAGE helped solvate insulin and that its lipid-extraction and fluidising effects promoted paracellular transport through the buccal tissue. This approach is not only attractive for delivery performance but also for future patch design. In the reported system, choline geranate was incorporated into a biodegradable polymeric buccal patch with a viscoelastic gel reservoir, and formulation optimisation identified a 70 vol% gel as a useful balance of viscoelastic behaviour, near-neutral surface pH, and mucoadhesion [29]. As summarised in Table 4, this system increased ex vivo transpor and produced measurable in vivo delivery, but long-term safety and broader translation acceptability remain incompletely defined. Supporting mechanistic work showed that choline geranate can preserve insulin’s secondary structure and reduce enzymatic degradation, although the study was not conducted in a buccal system [36]. Further studies are also needed to understand how insulin interacts with CAGE and how these interactions affect insulin stability, association state, and molecular conformation.

#### 3.3.3. Cell-Penetrating Peptides

Cell-penetrating peptide strategies were developed to improve buccal insulin uptake by strengthening insulin interaction with the epithelial barrier [28]. Figure 3 presents this concept. In this study, insulin was linked to low-molecular-weight protamine via PEG, and the active region of insulin was selectively protected before conjugation with dimethylmaleic anhydride to preserve bioactivity. The system was evaluated across TR146 multilayers, porcine buccal tissue, and rabbits to assess transport, permeation, and in vivo performance [28]. As summarised in Table 4, this approach demonstrated improved uptake and transport, with in vivo relative bioavailability reaching 26.86% and no visible signs of mucosal irritation [28].

Overall, these studies suggest that chemistry-led permeation strategies may be useful because they act more directly on insulin’s poor intrinsic permeability than residence-based systems alone. Hydrophobic ion pairing increases insulin lipophilicity, ionic liquids modify the local transport and stability environment, and cell-penetrating peptide conjugation promotes epithelial uptake. Their future value will depend on whether these transport gains can be translated into systems that are also stable, manufacturable, and suitable for repeated clinical use.

### 3.4. Device-Enabled Systems

Device-enabled systems differ from films, vesicles, and chemistry-led formulations by improving buccal delivery through mechanical assistance rather than relying solely on passive permeation enhancement. This is important because low intrinsic epithelial permeability remains a major barrier to translation [32]. Within this approach, the spray system is the primary traditional human proof of concept, while microneedles and suction-based patches represent the strongest recent attempts to overcome the passive permeability ceiling. Table 5 summarises the main device-enabled strategies relevant to buccal insulin delivery, including their delivery mechanism, relevance to insulin delivery, and the key translational challenges that remain.

#### 3.4.1. Buccal/Oral Insulin Spray System

Buccal and oral spray systems provide the clearest early human evidence that insulin can be absorbed through the oral mucosa. In the Rapid Mist device or Oral-Lyn platform, insulin is delivered as a high-velocity aerosol to the oropharyngeal cavity for transmucosal absorption, where part of the dose is absorbed transmucosally rather than swallowed [13,14,15,17]. Clinical studies showed faster absorption and earlier metabolic action than subcutaneous regular insulin. In healthy volunteers, the spray reached maximum serum insulin concentration at about 23 min, compared with about 83 min for subcutaneous insulin, while the maximum glucose infusion response occurred at about 44 min, compared with about 100 min [13]. A later study in type 1 diabetes patients also showed a dose–response relationship, with 5-, 10-, and 20-puff doses producing dose-dependent increases in insulin exposure while maintaining a rapid onset profile [14]. In a study of 18 patients with type 1 diabetes, buccal insulin spray administration produced postprandial glucose, insulin, and C-peptide responses generally comparable to those following subcutaneous insulin injection. Insulin concentrations were higher and the glucose-lowering effect more persistent at 90 and 120 min following subcutaneous administration. Buccal insulin did, however, produce peak plasma concentrations within around 30–60 min. [15]. Despite this clinical relevance, spray systems remained limited by high dose burden, short duration, and variable performance, so they are best viewed as important proof of concept rather than a complete translational solution.

#### 3.4.2. Buccal Microneedle Systems

Microneedle systems improve buccal insulin delivery by bypassing the epithelial barrier rather than relying on transport across intact mucosa [30]. Figure 3 presents this concept. This makes them especially relevant, as poor intrinsic permeability remains one of the main challenges in passive buccal delivery. In one study, a buccal microneedle patch localised about 1 mg of insulin in needle tips, delivered it within 30 s in swine, and produced measurable systemic exposure together with glucose lowering. Insulin reached maximum plasma concentration at about 40 to 50 min, showing that barrier bypass could support relatively rapid systemic delivery even without conventional injection [30]. The same study also included a human acceptability assessment of a sorbitol-based microneedle patch in 100 volunteers, where pain scores were low, the hard palate was the preferred application site, and 95% of participants said they would prefer this system over a conventional hypodermic injection. Although this human acceptability assessment did not administer insulin or evaluate its pharmacokinetics. Together, these findings make buccal microneedles one of the most promising recent device-based approaches, combining rapid delivery, direct barrier bypass, and early evidence of usability. The main remaining limitations are storage stability, manufacturing control, and the need for stronger repeat-use safety data [30,32].

#### 3.4.3. Suction and Octopus-Inspired Patch Systems

A newer class of device-enabled buccal systems improves peptide delivery through suction and tissue deformation rather than needle penetration. As presented in Figure 3, these octopus-inspired patches create local vacuum pressure that pulls, stretches, and thins the buccal mucosa, increasing the effective surface area for drug transport [31,32,44]. In one study, a mould-cast silicone suction patch with a lyophilised drug matrix increased effective surface area more than ninefold and achieved about 14.6% absolute bioavailability for teriparatide in beagle dogs, while also improving semaglutide delivery compared with an oral tablet [31]. Although this study did not test insulin, it still gives a useful idea for future buccal insulin design. Krupke et al. [44] Reported findings from an in vivo study in beagle dogs: a dose-adjusted semaglutide exposure increased 7- to 9-fold compared with the commercial oral tablet after 10 min of application, and bremelanotide achieved a relative bioavailability of 26% versus subcutaneous dosing after 20 min. These results suggest that biodegradable mechanical buccal systems could be useful for future insulin delivery, although insulin-specific evidence remains lacking and concerns remain about variability, dissolution under low-saliva conditions, and storage stability. Overall, the findings summarised in Table 5 suggest that device-enabled systems may offer a more practical way to overcome the buccal barrier.

## 4. Stability Constraints in Buccal Insulin Formulations

Preserving insulin stability is as important as increasing permeability, mucoadhesion, or mucosal residence time in buccal delivery systems. Many formulations are designed to enhance epithelial transport and mucosal contact, but if insulin loses its native structure during formulation, storage, administration, or mucosal residence, these gains become far less meaningful [2,6]. A successful buccal insulin system must therefore not only localise insulin at the absorption site, but also protect it from unfolding, aggregation, enzymatic degradation, and destabilising formulation microenvironments long enough for absorption to occur.

**Figure 4:**
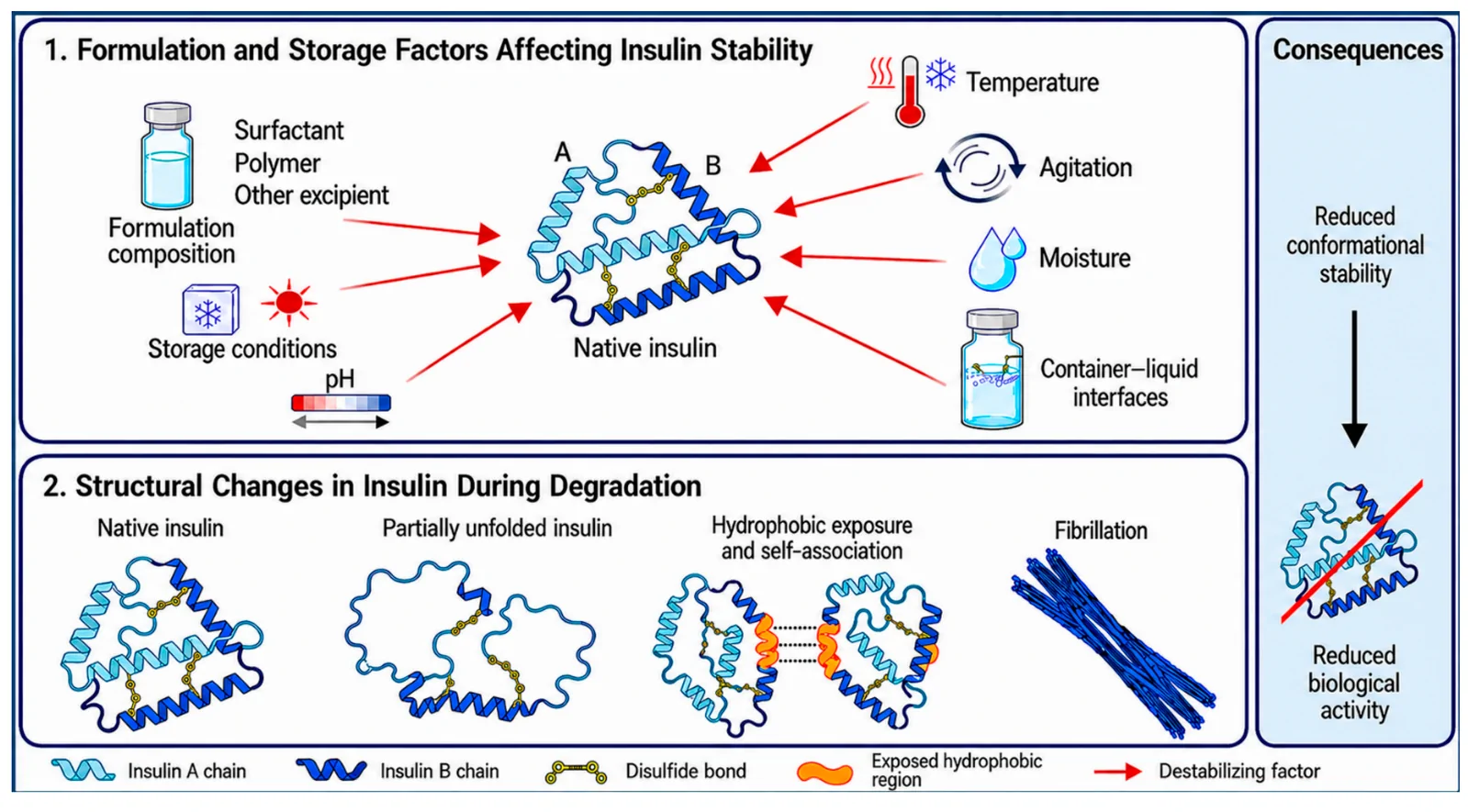
Schematic overview of formulation- and storage-induced insulin degradation. Source: Figure created using Figure Labs, AI Scientific Figure Illustrator (https://www.figurelabs.ai/, accessed 30 July 2026).

Human insulin consists of two chains linked by disulfide bonds, with 21 amino acids in the A chain and 30 in the B chain [33,45]. Its native conformation contains important *α*-helical regions that contribute to biological activity, receptor recognition, and structural stability [34,45]. Under destabilising conditions, insulin can partially unfold, expose hydrophobic regions, and progress through self-association, aggregation, and fibrillation, with loss of biological activity following these structural changes [33,34,35]. Figure 4 illustrates this concept. For these reasons, stability-related limitations in buccal insulin formulations should be evaluated at three distinct levels: biological instability following administration, physical instability during formulation and storage, and chemical instability associated with the formulation microenvironment. As illustrated in Figure 4, insulin is subjected to multiple interconnected formulation, storage, and physiological stresses during buccal delivery, many of which compromise its structural stability, reduce epithelial permeation, and ultimately limit systemic bioavailability.

### 4.1. Biological Instability After Dosing

As illustrated in Figure 2, biological instability begins as soon as insulin enters the oral cavity. Even when a formulation improves mucosal contact or permeability, insulin is still exposed to salivary dilution, enzymatic degradation at the mucosal surface, and limited residence time before part of the dose is swallowed or cleared away [2,6]. Together, these factors reduce the amount of structurally intact insulin available for absorption. Successful buccal systems must therefore do more than deliver insulin to the mucosa; they must also protect it long enough to allow for meaningful uptake.

### 4.2. Physical Instability During Formulation and Storage

Physical instability is another major concern in buccal insulin delivery because insulin can lose activity during preparation and storage through unfolding, aggregation, precipitation, or carrier destabilisation. In chitosan-based mucoadhesive buccal films, insulin structure was better preserved when L-arginine was included, and drying at 2 to 8 °C retained more insulin than drying at room temperature, showing that stability depends not only on the carrier but also on how the dosage form is processed [19]. A similar point was seen in a deformable nanovesicle-loaded gelatine gel system, where the gel matrix helped preserve vesicle morphology, particle size, pH, and insulin content for 3 months at 4 °C, whereas less stabilised liquid systems were more prone to leakage, fusion, or precipitation [26]. Together, these findings show that physical instability in buccal insulin systems is not limited to insulin itself but also involves the structural integrity of the surrounding dosage form [19,26].

### 4.3. Chemical Instability and Formulation Environment

Chemical instability further complicates buccal insulin delivery because insulin can be easily affected by unfavourable pH conditions, reactive formulation components, surfactants, and prolonged exposure to moisture-rich environments. Figure 4 illustrates this concept. This means that excipient choice and the local formulation environment are just as important as carrier design. Banerjee et al. [36] reported that choline geranate preserved the characteristic insulin *α*-helical circular dichroism profile during storage, suggesting that the formulation environment helped maintain insulin close to its native folded state. The same study also showed reduced enzymatic degradation and suggested that limiting insulin–water interactions may help reduce hydrolysis and self-aggregation. Although this was not a buccal study, it remains highly relevant because it shows how strongly the local chemical environment can influence whether insulin stays structurally intact long enough for a delivery system to work. Table 6 summarises the main stability-related constraints and their formulation implications. As described previously, stability in buccal insulin delivery is not limited to a single formulation problem but reflects a set of related constraints that act before, during, and after administration. It shows that insulin can be compromised by biological factors after dosing, including salivary dilution, enzymatic degradation, and short mucosal residence time; by physical instability during formulation and storage, such as unfolding, aggregation, precipitation, or carrier destabilisation; and by chemical effects arising from the formulation environment itself, including nonoptimal pH, excipient interactions, and prolonged exposure to hydrated systems. Taken together, these parameters show that successful buccal insulin delivery depends not only on improving permeation but also on preserving insulin in a structurally intact and pharmaceutically usable form throughout the full delivery pathway.

Insulin is essential for glucose regulation through activation of the insulin receptor and remains the mainstay of diabetes treatment. However, its therapeutic use is challenged because of structural instability, including unfolding and formation of cross-β-sheet amyloid fibrils. The limited understanding of the molecular basis of insulin fibrillation has hindered the development of effective strategies to prevent it [45]. In the same study, the authors investigated insulin fibrils using cryo–electron microscopy (cryo-EM). This study identified several fibril architectures composed of protofilaments containing both insulin A and B chains connected by disulfide bonds. A two-protofilament fibril was resolved at 3.2 Å, revealing the β-sheet organisation and inter-protofilament interactions that stabilise the fibril. Guided by this structure, the researcher designed insulin variants with reduced fibrillation tendency while retaining native IR (insulin receptor) signalling activity, highlighting their potential as more stable therapeutic candidates for type 1 diabetes [45].

## 5. Translational Prospects for Future Buccal Insulin Delivery

One of the main challenges in the buccal insulin literature is the lack of standardised benchmarks for comparing systems across studies. Different approaches use different formulations, models, doses, and outcome measures, making direct comparison difficult and potentially obscuring true translational value. Future progress will therefore depend not only on better delivery results, but also on clearer criteria for judging which systems are most likely to translate successfully.

Buccal insulin delivery remains an attractive goal because it offers a noninvasive route with direct access to a vascularized mucosa while avoiding gastrointestinal degradation and hepatic first-pass metabolism [2,6]. The main question is no longer whether buccal insulin can work in principle, but whether it can be developed into a system that is stable, dose-efficient, reproducible, and clinically practical. This is especially important because the field already contains real proof of concept. Human clinical studies of oral/buccal insulin sprays provide evidence consistent with transmucosal insulin absorption. Cernea et al. [13,14] evaluated oral insulin spray in healthy volunteers and patients with type 1 diabetes, respectively, whereas Pozzilli et al. [15] specifically investigated buccal spray insulin in patients with type 1 diabetes. All three studies involved human in vivo administration and measured circulating insulin and/or glucose-lowering responses. However, later reappraisal showed that these early systems were limited by low relative biopotency, high dose burden, and substantial variability [17]. These findings remain important because they established route feasibility in humans, but they also set a practical benchmark that newer systems still need to exceed. Model selection also deserves particular attention in preclinical studies. Reported permeability findings for insulin differ substantially across systems, with log permeability coefficients of about −8.18 in porcine buccal tissue and about −5.92 to −7.10 in TR146 models [16]. Secondly, a formulation that performs well in a more permissive model may therefore not perform as strongly under more realistic buccal conditions. Permeability should also not be judged alone, because insulin transport is influenced simultaneously by epithelial resistance, small pore size, salivary washout, mucus, and enzymatic degradation [6,16,39]. Additionally, as mentioned in Table 1, buccal insulin delivery is limited by several barriers acting simultaneously rather than by permeability alone. Insulin transport across the buccal mucosa also depends on its association state. Insulin can exist as individual molecules or larger aggregates such as dimers and hexamers. These forms can differ in size and diffusivity. Therefore, formulation factors such as pH, insulin concentration, zinc, and other excipients may influence both insulin stability and its transmucosal absorption [41]. When designing new formulations for buccal insulin delivery, these constraints should be considered collectively.

Different formulation classes contribute in different ways, and each is useful for a particular part of the translational problem. Mucoadhesive films and patches, summarised in Table 2, are most useful for improving residence and supporting directional release at the buccal surface. According to a bilayer film study, which used an ethyl cellulose backing layer with an adhesive HPMC-PAA interpolymer complex layer, it showed prolonged adhesion, a 2.24-fold increase in insulin AUC relative to free insulin, and glucose control for about 8 h in diabetic rats [18]. In another study, an insulin-loaded chitosan film was prepared by solvent casting with glycerin and L-arginine, retaining at least 80% of the theoretical insulin load and producing measurable glucose lowering, although it remained weaker than subcutaneous insulin [19]. These findings show that film and patch systems can improve buccal residence and support the value of improved film engineering in buccal insulin delivery, although they may remain dose inefficient.

Nanocarrier-based systems, compared in Table 3, contribute more than residence alone because they can combine insulin protection, controlled release, and carrier–mucosa interaction. In one study, insulin-loaded PEG-b-PLA nanoparticles were incorporated into Chitosan films with about 71% encapsulation efficiency [21] while another study showed measurable buccal flux from nanoparticle film systems [22]. As summarised in Table 3, deformable vesicle systems appear to be a promising passive nanocarrier approach for buccal insulin delivery. Across several preclinical studies, their flexible phospholipid-based structures were associated with improved delivery performance [23,24,26]. There is one study that also reports that incorporating a similar vesicular system into a gelatine-based matrix helped improve storage stability for 3 months at 4 °C in a gelatine matrix [26]. Taken together, these findings suggest that deformable vesicle-based systems may be particularly useful when insulin protection, transport enhancement, and formulation stability need to be addressed within the same platform. However, despite these promising findings, further work is needed to ensure adequate storage stability, reproducible performance, and a formulation process that is practical for clinical use.

Chemistry-led systems, summarised in Table 4, are particularly useful because they address poor intrinsic insulin permeability more directly. In hydrophobic ion pairing with bile salt derivatives, permeability increased about 3-fold in TR146 cells and about 51.76-fold ex vivo, showing how changes in insulin lipophilicity can strongly affect transport [27]. Choline geranate buccal patch increased ex vivo permeation to about 7-fold and produced measurable in vivo bioavailability [29]. In another study, the PEG-linked low-molecular-weight protamine conjugate is also notable because it achieved in vivo relative bioavailability up to 26.86% [28]. These findings show that chemistry-led approaches may be especially useful for solving the permeability problem more directly, but often at the cost of greater molecular complexity, less certain safety definition, and more difficult translation. Across these formulation approaches, insulin is combined with a range of excipients, permeation enhancers, bile salts, and carrier materials. However, the effects of these components, individually and in combination, on insulin stability and structural properties remain insufficiently understood.

Device-enabled systems, as highlighted in Table 5, remain particularly important because they reduce dependence on passive epithelial transport, which continues to limit buccal insulin delivery. Spray-based systems still provide the clearest human proof of concept for rapid buccal and oral insulin delivery [13,14,15]. In human studies, oral insulin spray reached an insulin *T_max_* of about 23 to 30 min, compared with about 83 min for subcutaneous regular insulin, indicating a faster onset that may be relevant for prandial use [13,15,17]. However, the latter advantage is limited by low dose efficiency, high burden, and variability [17]. The microneedle patch is also notable because it localised about 1 mg of insulin in the needle tips, delivered the dose within 30 s, produced measurable systemic exposure in swine, and was supported by a favourable acceptability data study in 100 volunteers [30]. Studies of suction-based patches further show that mechanical tissue deformation and increased contact area can improve peptide delivery without needle penetration [30,44]. Taken together, these findings support device-enabled approaches as highly relevant for future insulin translation, although their practical value will still depend on repeat-use safety, storage stability, usability, dose consistency, and overall product feasibility [30,32,44].

For future studies, the key issue is not whether a system improves one result, but whether it performs well across the factors that matter for translation. These include preservation of insulin integrity, dose efficiency, clinically interpretable pharmacokinetic and pharmacodynamic performance, realistic mucosal residence, credible model selection, local safety, manufacturability, storage practicality, and user acceptability.

## 6. Conclusions

Insulin delivery via the buccal route has been investigated through a range of formulation and device-based approaches. Earlier human studies have confirmed that insulin can be absorbed through the oral mucosa and can produce relatively rapid systemic effects, but they have also highlighted persistent limitations, including low dose efficiency, variability, and incomplete control over exposure. Subsequent advances expanded the field but did not fully resolve these challenges. Mucoadhesive films and patches improved residence time and directional release; nanocarrier-based and deformable vesicle systems supported insulin protection and transport; chemistry-led strategies addressed poor intrinsic permeability more directly; and device-enabled platforms suggested that mechanical assistance may be needed when the intact buccal mucosa imposes a strong permeability barrier. Despite this progress, no current platform yet satisfies the full requirements for clinical translation. The central challenge is no longer whether insulin can be delivered through the buccal route, but whether a system can preserve insulin stability, provide reproducible therapeutic exposure, maintain local tolerability, and remain manufacturable and storage-stable within a single product concept. Future progress will therefore depend on integrated platform designs in which permeation, stability, pharmacokinetic and pharmacodynamic performance, safety, residence time, and product feasibility are addressed together rather than as separate formulation goals.

## Figures and Tables

**Table 1:** Overview of barriers to buccal insulin delivery.

Barrier Type	Barrier	Effect	Key Feature	References
Physiochemical	High molecular weight and hydrophilicity	Limits absorption and permeation	Human insulin is a 51-amino-acid peptide hormone with a molecular weight of approximately 5808 Da	[2,38]
Physical	Stratified buccal epithelium	Restricts diffusion	Human buccal epithelium is about 500 to 600 μm thick and consists of 40 to 50 cell layers	[39]
	Intercellular lipid domains and tight junctions	Reduce transport	Buccal lipid domains and intercellular junctions restrict passive permeation of hydrophilic macromolecules	[6,16,39]
	Small effective pore radius	Limits paracellular passage	The estimated effective pore radius is about 1.5 to 3 nm	[16]
	Low intrinsic permeability	Limits passive permeation	Log permeability coefficient is about −8.18 in porcine buccal tissue and −5.92 to −7.10 in TR146 models	[16]
Physiological	Salivary washout	Reduces residence time	Dilutes the drug and shortens the residence time	[6,9,39]
	Mucus barrier	Restricts epithelial access	Mucin forms a gel barrier that can restrict diffusion and entrap insulin before epithelial contact	[6,39,40]
Enzymatic	Peptidases in saliva and mucosa	Promotes insulin degradation	Includes aminopeptidases, carboxypeptidases, endopeptidases, and DPP-IV (Dipeptidyl peptidase)	[2,39]

**Table 2:** Mucoadhesive film and patch strategies for buccal insulin delivery.

Experimental Approach	Purpose of the Study	Preparation Method	Characterisation Methods	Main Findings	Limitation	References
Double-layer mucoadhesive film with HPMC-PAA interpolymer complex, insulin, protamine, and ethyl cellulose backing	To improve long-term mucoadhesion, support unidirectional release, and enhance insulin penetration	Sequential coating with EC backing layer followed by IPC adhesive layer	IPC optimisation, viscosity and turbidity testing, adhesion force and adhesion time, release studies, pharmacokinetics, in vivo glucose evaluation (diabetic rat model)	Adhesion force 120.2 ± 20.3 N/m^2^, adhesion duration 491 ± 45 min, insulin AUC 2.24-fold higher than free insulin administered by subcutaneous injection, glucose control maintained for about 8 h in T1DM diabetic rats	Provides preclinical evidence from ex vivo tissue and T1DM rates; clinical translation was not evaluated	[18]
Chitosan-based mucoadhesive buccal film containing insulin, glycerin, and L-arginine	To provide a flexible, noninvasive film with acceptable insulin loading and buccal pharmacological activity	Film casting from a chitosan-based film-forming solution, followed by drying and unit-dose preparation	Film appearance and flexibility, insulin content by ELISA, spectroscopic analysis, and in vivo glucose-lowering study (male Sprague-Dawley rats were used for in vivo evaluation)	Films retained at least 80% of the theoretical insulin load, reduced blood glucose up to 40% during the first 8 h, and achieved about 57% of the subcutaneous insulin effect	Performance remained below subcutaneous insulin, and formulation properties were sensitive to composition	[19]
Triple-layer unidirectional mucoadhesive buccal tablet concept	To reduce salivary loss, improve directional delivery, and support a dry peptide dosage form	Conceptual multilayer design with insulin nanoparticles converted to dry powder and incorporated into an active layer with mucoadhesive and backing layers	Conceptual design, layer configuration, and polymer selection	Suggested the approach of combining nanoparticle loading, dry-state formulation, mucoadhesion, and unidirectional release in one system	The concept was supported by limited reported in vivo validation for buccal insulin	[20]

**Table 3:** Nanocarrier-based strategies for buccal insulin delivery.

Experimental Approach	Composition	Method Used	Key Findings	References
Nanoparticle-loaded buccal film	Insulin-loaded PEG-b-PLA nanoparticles in chitosan film	Double emulsion solvent evaporation for nanoparticles, followed by solvent casting for films	Optimised nanoparticles showed a size of 215.5 ± 2.5 nm and an encapsulation efficiency of about 71%; films showed homogeneous distribution. The insulin-loaded nanoparticles showed biphasic release at 6 h, followed by sustained release for 5 weeks.	[21]
Nanoparticle-based buccal film	Insulin-coated nanoparticles with D, L-valine core in ERL or ERL-HPMC film	Antisolvent co-precipitation for nanoparticles, followed by solvent casting	Optimised ERL film showed about 65% insulin release in 4 h and a flux of 0.34 ± 0.05 μg/h/cm^2^ through a human buccal mucosa model (Tridimensional human buccal tissue (EpiOral^®^, Mattek Corporation, Ashland, MA, USA).	[22]
Deformable vesicles	SPC and sodium deoxycholate deformable vesicles loaded with insulin	Reverse phase evaporation followed by hydration and sonication	Relative pharmacological bioavailability 15.59% and relative bioavailability 19.78%, higher than conventional vesicles. Bioavailability values were calculated relative to subcutaneous insulin solution (for in vivo study, a fasted male rabbit model was used); no ex vivo permeability study performed.	[23]
Deformable nanovesicles	Insulin–phospholipid complex nanovesicles with lecithin, Tween 20, and sodium deoxycholate	Thin-film hydration	Optimised formulation showed particle size of 85.84 ± 2.38 nm and encapsulation efficiency of 77.47 ± 3.85%; ex vivo insulin permeation was evaluated using porcine buccal mucosa mounted in Franz diffusion cells. In a separate in vivo rabbit study, IPC-DNVs showed a relative pharmacological bioavailability of 15.53%.	[24]
Elastic liposomes	Insulin-loaded soy lecithin liposomes with SC or SGDC as edge activator	Thin-film hydration followed by extrusion	Optimised SGDC liposomes showed a size of 150.40 ± 3.50 nm, EE of 79.41 ± 5.07%, and a 4.33-fold higher permeability coefficient than insulin solution in ex vivo porcine buccal mucosa mounted in Franz diffusion cell study. No in vivo study conducted.	[25]
Gel-stabilised deformable vesicles	Insulin–phospholipid complex deformable nanovesicles in 2% gelatine matrix	Thin-film hydration and high-pressure homogenization, followed by mixing into gelatine gel	Stable at 4 °C for 3 months with preserved delivery performance; in vivo relative pharmacological bioavailability 14.90 ± 3.12% measured in rabbit (TR146 human buccal epithelial cell layers model was used to study in vitro uptake and transport model).	[26]
Elastic bilosomes	Insulin-loaded elastic bilosomes modified with cholic acid derivatives	Vesicle preparation by thin-film hydration and TR146 cell permeation study	Showed facilitated insulin permeation across TR146 cells, supporting bile salt-modified flexible carriers as a transport-enhancing approach (TR146 human buccal epithelial cell layer model was used for in vitro permeability study).	[42]

**Table 4:** Comparison of chemistry-led permeation strategies for buccal insulin delivery.

Approach	Study Rationale	Model Used	Key permeability or Bioavailability Findings	Key Limitation	References
Hydrophobic ion pairing	To increase insulin lipophilicity and improve membrane transport	Multilayer TR146 cells and ex vivo porcine buccal tissue were used for permeability study	Optimised complex produced about 3-fold higher permeability in TR146 cell model study and about 51.76-fold increase in permeability coefficient across ex vivo porcine buccal tissue	No in vivo validation and no integrated buccal dosage form was evaluated	[27]
Ionic liquid system	To enhance buccal transport while preserving insulin activity during patch preparation	Ex vivo porcine buccal tissue and in vivo study in rat	Choline geranate patch increased ex vivo insulin transport approximately sevenfold. The GAGE/insulin patch produced a relative bioavailability of about 5.4 ± 0.79% in an in vivo study.	Long-term safety, clinical translation, and patient acceptability not evaluated	[29]
Ionic liquid mechanistic support	To stabilise insulin and reduce degradation while improving transport-related behaviour	In vitro model: Caco-2 human intestinal epithelial cell monolayers. Adult, non-diabetic male Wistar rat	Choline geranate preserved insulin structure, reduced proteolysis, and enhanced paracellular transport, oral insulin exposure and glucose-lowering activity	Not a buccal study (oral and intrajejunal delivery)	[36]
Cell-penetrating peptides	To improve epithelial interaction and buccal uptake of insulin	Ex vivo Porcine buccal mucosa, multilayer TR146 human buccal epithelial model, and for in vivo study rabbit buccal model	In vivo study: male Japanese rabbit; pharmacological bioavailability was 21.34% based on glucose lowering, whereas relative bioavailability was up to 26.86% based on serum insulin exposure; both were relative to subcutaneous insulin at a 10 IU/kg buccal dose	Chemical conjugation increases formulation complexity, and clinical translation was not evaluated	[28]

**Table 5:** Device-enabled strategies relevant to buccal insulin delivery.

Device-Enabled Technique	Main Features of Enhancing Delivery	Relevance to Buccal Insulin Delivery	Main Translational Challenge	References
Oral/Buccal spray	Human pharmacokinetic and pharmacodynamic proof of concept	Shows rapid prandial-type insulin delivery in humans, with insulin *T_max_* about 23 to 30 min	Low bioavailability, high dose burden, and variable performance across users	[13,14]
Buccal microneedle patch	Direct barrier bypass with strong acceptability data	Supports relatively rapid systemic insulin delivery by barrier bypass, with insulin *T_max_* about 40 to 50 min in swine	Stability during storage, manufacturing control, and repeat-use safety still need stronger findings	[30,32]
Suction or octopus-inspired patch	Noninvasive mechanical enhancement of mucosal transport	Shows that epithelial stretching and increased contact area may improve peptide delivery without needle penetration, which is attractive for future insulin systems	No insulin-specific in vivo evidence is available yet, and the required application time remains relatively long	[31,32]
Biodegradable suction patch	The suction patch concept while preserving peptide delivery performance	This suggests that future buccal peptide devices may need to consider not only efficacy, but also material design suitable for repeated long-term use	Insulin-specific evidence is still lacking, and concerns remain about variability and storage stability	[44]

**Table 6:** Stability challenges and formulation implications in buccal insulin delivery.

Stability Aspect	Primary Factors Affecting Stability	Impact on Insulin or Dosage form	Implications for Buccal Formulation Performance	References
Post-administration biological instability	Salivary dilution, mucosal and salivary enzymes, short residence time, swallowing	Reduces the amount of structurally intact insulin available at the absorption site	This can limit absorption even when the formulation shows strong mucoadhesion or permeation enhancement	[2,6]
Native conformational instability	Heat, agitation, acidic pH, interfacial stress, and excipient-induced conformational disturbance	Promotes partial unfolding, exposure of hydrophobic regions, self-association, and fibrillation	Structural disruption reduces biological activity and compromises formulation performance	[33,34,35,45]
Processing-induced instability	Drying conditions, polymer–protein interactions, and thermal or mechanical stress during manufacturing	Can reduce insulin content or disrupt ordered secondary structure during dosage form preparation	Processing conditions affect whether insulin remains stable and usable in buccal films	[19]
Storage-related physical instability	Vesicle fusion, leakage, precipitation, matrix relaxation, and carrier destabilisation in hydrated systems	Alters particle size, morphology, pH, and retained insulin content during storage	Poor storage stability reduces shelf-life, reproducibility, and translational feasibility	[26]
Chemical instability associated with formulation environment	Nonoptimal pH, reactive hydrated microenvironments, prolonged water exposure, and excipient incompatibility	Can promote hydrolysis, aggregation, or loss of native helical structure	The formulation microenvironment is critical for preserving insulin bioactivity during storage and delivery	[33,36]

## Data Availability

No new data were created or analysed in this study. Data sharing is not applicable to this article.

## Abbreviations

The following abbreviations are used in this manuscript:

AUC	area under the curve
DPP-IV	dipeptidyl peptidase IV
EC	ethyl cellulose
EE	encapsulation efficiency
ELISA	enzyme-linked immunosorbent assay
ERL	Eudragit RLPO
FTIR-MS	Fourier-transform infrared micro spectroscopy
FITC	fluorescein isothiocyanate
GLP-1	glucagon-like peptide 1
HPMC	hydroxypropyl methylcellulose
Lipo	liposome
ICNP	insulin-coated nanoparticle
IPC	interpolymer complex
PEG	polyethylene glycol
PAA	polyacrylic acid
PEG-b-PLA	polyethylene glycol-block-poly lactic acid
PDI	polydispersity index
RBA	relative bioavailability
RPB	relative pharmacological bioavailability
SC	sodium cholate
SGDC	sodium glycodeoxycholate
SPC	soybean phosphatidylcholine
SRCD	Synchrotron Radiation Circular Dichroism
TEER	transepithelial electrical resistance
T1DM	type 1 Diabetes Mellitus.
TR146	human buccal carcinoma cell line model
Tmax	time to maximum concentration
